# Root exudate fingerprint of *Brachiaria humidicola* reveals vanillin as a novel and effective nitrification inhibitor

**DOI:** 10.3389/fmolb.2023.1192043

**Published:** 2023-12-05

**Authors:** Konrad Egenolf, Jochen Schöne, Jürgen Conrad, Christina Braunberger, Uwe Beifuß, Jacobo Arango, Frank Rasche

**Affiliations:** ^1^ Institute of Agricultural Sciences in the Tropics (Hans-Ruthenberg-Institute), University of Hohenheim, Stuttgart, Germany; ^2^ Tropical Forages Program, The Alliance of Bioversity International and the International Center for Tropical Agriculture (CIAT), Cali, Colombia; ^3^ Institute of Phytomedicine, University of Hohenheim, Stuttgart, Germany; ^4^ Institute of Chemistry, University of Hohenheim, Stuttgart, Germany

**Keywords:** *Urochloa humidicola* (Poaceae), biological nitrification inhibition (BNI), forages, allelopathy, phenolics, *Nitrosomonas europaea*

## Abstract

**Introduction:** Biological Nitrification Inhibition (BNI) is defined as the plant-mediated control of soil nitrification via the release of nitrification inhibitors. BNI of *Brachiaria humidicola* (syn. *Urochloa humidicola*) has been mainly attributed to root-exuded fusicoccane-type diterpenes, e.g., 3-*epi*-brachialactone. We hypothesized, however, that BNI of *B. humidicola* is caused by an assemblage of bioactive secondary metabolites.

**Methods:** *B. humidicola* root exudates were collected hydroponically, and metabolites were isolated by semi-preparative HPLC. Chemical structures were elucidated by HRMS as well as 1D and 2D NMR spectroscopy. Nitrification inhibiting potential of isolated metabolites was evaluated by a *Nitrosomonas europaea* based bioassay.

**Results and discussion:** Besides previously described brachialactone isomers and derivatives, five phenol and cinnamic acid derivatives were identified in the root exudates of *B. humidicola*: 2-hydroxy-3-(hydroxymethyl)benzaldehyde, vanillin, umbelliferone and both *trans-* and *cis*-2,6-dimethoxycinnamic acid. Notably, vanillin revealed a substantially higher nitrification inhibiting activity than 3-*epi*-brachialactone (ED_50_ ∼ 12.5 μg·ml^−1^, ED_80_ ∼ 20 μg·ml^−1^), identifying this phenolic aldehyde as novel nitrification inhibitor (NI). Furthermore, vanillin exudation rates were in the same range as 3-*epi*-brachialactone (1–4 μg·h^−1^·g^−1^ root DM), suggesting a substantial contribution to the overall inhibitory activity of *B. humidicola* root exudates. In relation to the verification of the encountered effects within soils and considering the exclusion of any detrimental impact on the soil microbiome, the biosynthetic pathway of vanillin via the precursor phenylalanine and the intermediates *p*-coumaric acid/ferulic acid (precursors of further phenolic NI) might constitute a promising BNI breeding target. This applies not only to *Brachiaria spp*., but also to crops in general, owing to the highly conserved nature of these metabolites.

## 1 Introduction

Perennial grasses have been described to control soil nitrification (Theron, 1951; Sylvester Bradley et al., 1988; Lata et al., 2004), an attribute contributing to the mitigation of nitrification related N losses, including NO_3_- leaching and N_2_O emissions (Subbarao et al., 2009; Byrnes et al., 2017). Biological Nitrification Inhibition (BNI), defined as the plant-exerted control of nitrifiers through the release of allelochemicals (Subbarao et al., 2006), is one mechanism explaining the reduction of soil nitrification rates (Coskun et al., 2017; Nardi et al., 2020). Several plants, including mostly graminaceous crops, have been screened for nitrification inhibiting secondary metabolites in plant tissue and especially root exudates (Coskun et al., 2017; Lu et al., 2021; Otaka et al., 2023). Notably, the tropical forage grass *Brachiaria humidicola* (syn*. Urochloa humidicola*) has been acknowledged to effectively control soil nitrification (Sylvester Bradley et al., 1988; Karwat et al., 2018; Nuñez et al., 2018). In *B. humidicola*, BNI has been attributed to different phenolic compounds, e.g., methyl coumarate and methyl ferulate, which are released during root turnover (Gopalakrishnan et al., 2007), as well as different fusicoccane-type diterpenes called brachialactones, which are actively exuded into the rhizosphere (Subbarao et al., 2009; Egenolf et al., 2020; Egenolf et al., 2021). It must be noted, however, that these nitrification inhibiting compounds have been determined solely under artificial conditions (hydroponics), while the evidence of their presence and activity in the rhizosphere or bulk soil of prevalent *B. humidicola* stands remains elusive. In fact, preliminary studies aiming at the *in situ* quantification of exudation and accumulation of the mentioned nitrification inhibitors (NI) in the rhizosphere of soil-grown *B. humidicola* plants via the sorption-filter technique described by Neumann et al. (2014) and different soil extraction approaches, were not successful (Egenolf, unpublished data). This may be attributed to well-known technical challenges during both *in situ* root exudate collection as well as soil extraction approaches (White, 1991; Neumann et al., 2009). On the other hand, it could be also ascribed to the overall low internal tissue concentrations of methyl coumarate, methyl ferulate (G. Subbarao, personal communication), and 3-*epi*-brachialactone [2–8 μg·g^−1^ root dry matter (DM) (Egenolf et al., 2021)], as well as low brachialactone and 3-*epi*-brachialactone exudation rates [0.4–4.0 μg·h^−1^·g^−1^ root DM (Subbarao et al., 2009; Egenolf et al., 2021)]. With this, the involvement of yet undiscovered active ingredients or alternative modes of action could be suggested. Consequently, complementary theories on soil nitrification control are broadly discussed, including microbial N immobilization resulting in the out-competition of ammonia oxidizers, as well as efficient uptake of NO_3_^−^ by plants (Vázquez et al., 2020; Teutscherová et al., 2021; Egenolf et al., 2022). Here, however, it was our main ambition to verify the existence of so far undiscovered NI. More precisely, we hypothesize that root exudates of *B. humidicola* contain a broad range of secondary metabolites with nitrification inhibiting activity. Thereby, the objective was to collect root exudates in a hydroponic system (limiting confounding effects of an associated soil microbiome), identify major secondary metabolites present in the root exudates of *B. humidicola* and evaluate their nitrification inhibiting activity.

## 2 Material and methods

### 2.1 Root exudate collection

*Brachiaria humidicola* cv. CIAT 679 plants (commercial name “Tully,” ranked as high BNI cultivar) were propagated and raised during 6 weeks in a growth chamber-based hydroponic system with a day length of 12 h (6:00–18:00 h), light intensity of 525 W·m^−2^, air humidity of 75% and day/night temperatures of 30/20°C. The nutrient solution contained (µM): NH_4_NO_3_ 1200, KNO_3_ 400, Ca(NO_3_)_2_ 400, HNO_3_ 600, K_2_HPO_4_ 200, MgSO_4_ 200, MgCl_2_ 100, Na_2_SiO_3_ 200, FeNa-EDTA 50, H_3_BO_3_ 10.0, MnSO_4_ 4.0, ZnSO_4_ 4.0, CuSO_4_ 1.0, Na_2_MoO_4_ 1.0. The nutrient solution was exchanged every 2–3 days and pH of fresh nutrient solution was adjusted at 4.8. Root exudate collection was performed into fresh nutrient solution with a plant density of 4 plants L^−1^ of nutrient solution during 24 h. After root exudate collection, the nutrient solution was filtered to remove root debris and organic compounds were extracted by liquid phase extraction (by that eliminating salts contained in the nutrient solution as well as any root-derived impurities, e.g., proteins). For that, sodium chloride was added to the nutrient solution (polar phase) until saturation, facilitating the subsequent extraction of organic compounds with 300 ml of ethyl acetate (organic phase) per 1 L of nutrient solution in a separating funnel. The ethyl acetate phase was filtered through a layer of 2 cm of anhydrous Na_2_SO_4_ to remove any remaining water. The extraction of the nutrient solution was repeated. The two ethyl acetate extracts were pooled, concentrated *in vacuo* and stored at 4°C until further analysis. In total, root exudates of approximately 400 plants were pooled to obtain ∼3 mg of raw exudate, yielding between 100 and 200 μg pure compounds after semi-preparative fractionation (next section).

### 2.2 Root exudate fractionation

For HPLC-PDA analysis and semi-preparative HPLC fractionation, ethyl acetate extracts were dried under a N_2_ flow (30°C) and resuspended in H_2_O/acetonitrile (1/1, v/v) or H_2_O/isopropanol/acetonitrile (1/1/1, v/v/v), respectively. Root exudates were screened for major secondary metabolites based on PDA chromatograms obtained via HPLC-PDA analysis at a wavelength range of 200–600 nm (Accela HPLC/LTQ Velos MS, Thermo Scientific, Waltham, United States) using a Kinetex 2.6 μm XB-C18 100A reverse phase column (Phenomenex, Torrance, United States) with formate buffer (10 mM, pH 3.7) as polar and acetonitrile as nonpolar eluent (flow rate 0.5 ml·min^−1^).

Subsequently, selected major secondary compounds were isolated via semi-preparative HPLC (Knauer Smartline, Berlin, Germany), using an EC 250/10 Nucleodur PolarTec 5 µm reverse phase column (Macherey-Nagel, Düren, Germany) in a first, and a xSelect HSS Prep T3 5 µm 10 mm × 150 mm reverse phase column (Waters, Milford, United States) in a second step. Both fractionation steps were conducted with 0.01% trifluoroacetic acid as polar and acetonitrile as nonpolar eluent (flow rate 5 ml·min^−1^). The applied eluent gradients are provided in the Supplementary Tables S1–S3 (Supplementary Material).

### 2.3 HRMS

In order to accurately assess the molecular mass of purified secondary compounds high resolution mass-spectra were recorded on a QExactive Plus Electrospray Mass Spectrometer (Thermo Fisher Scientific Waltham, United States) coupled to an Agilent 1290 Ultra Performance Liquid Chromatography System. Measurement parameters were applied according to the standard protocols of our institution: ESI positive, HESI Source, Capillary Temp 360°C, Sheath gas 60, Aux gas 20, Probe Heater 380°C, Full scan: 100–800 m/z, resolution 35.000, MS2: resolution 17.500, NCE 15, 25, 35. The eluent gradient is provided in Supplementary Table S4 (Supplementary Material).

### 2.4 NMR spectroscopy

For the elucidation of molecular structures NMR-spectra were recorded on an Avance HD III 600 MHz spectrometer, equipped with a BBO Prodigy cryo-probe (Bruker, Billerica, United States). Metabolites were dissolved in methanol-d_4_ in standard 5 mm NMR tubes or 2 mm MATCH NMR tubes. The ^1^H and ^13^C chemical shifts were referenced to the residual solvent signal at *δ*_H/C_ 3.35 ppm/49.0 ppm. HSQC, HMBC, NOESY, COSY and selective 1D-TOCSY spectra were recorded using standard Bruker pulse sequences at 298 K. For processing and evaluation of NMR spectra, the software SpinWorks 4.2.8.0 (Copyright 2017, K. Marat, University of Manitoba, Canada) was used.

### 2.5 Assessment of nitrification inhibiting potential

Nitrification inhibiting activity was assessed by means of the *Nitrosomonas europaea* based bioluminescence assay developed by Subbarao et al. (2006) and adjusted by Nuñez et al. (2018). Briefly, the bioluminescent *N. europaea* IFO 14298 (ATCC 19178) pHLUX20 strain developed by Iizumi et al. (1998) was cultured in a kanamycin (50 μg·ml^−1^) supplemented phosphate buffered growth medium for 6 days at 50 rpm and 28°C. The growth medium was composed of (mM): KH_2_PO_4_ 5.14, Na_2_HPO_4_ 95.1, (NH_4_)_2_SO_4_ 18.91, NaHCO_3_ 5.95, CaCl_2_ 0.034, MgSO_4_ 0.041, Fe (III) EDTA 0.0027. Two hundred ml of liquid culture were centrifuged at 2,500 g for 20 min and the *N. europaea* pellet was resuspended in 50 ml of fresh culture medium. Compounds to be tested were dissolved in 1 µl DMSO, diluted to 100 µl with distilled H_2_O and then incubated with 125 μl *N. europaea* culture for 15 min (900 rpm, 15°C) prior to bioluminescence measurements. Bioluminescence was measured on 100 µl aliquots with two technical replicates on a Glomax 20/20 (Promega, Fitchburg, United States) integrating the flash luminescence reaction 2–10 s after automated injection of 25 µl of decanal (1%) in ethanol. Every measurement was repeated with three biological replicates and inhibition was calculated relative to the DMSO blank.

### 2.6 Assessment of vanillin exudation in dependence of rhizosphere pH and nutritional N-form

Root exudation of vanillin by *B. humidicola* cv. CIAT 679 (commercial name “Tully”) was assessed as described by Egenolf et al. (2021). In brief, exudation patterns were investigated in a two-factorial hydroponic experiment. For factor “pH,” the pH of the trap solution was adjusted to target values of 4.2 and 6.8 by addition of HCl and Na_2_CO_3_, respectively. Factor “Trap solution” consisted of a NH_4_^+^ and NO_3_^−^ treatment [see Experiment 1 in Egenolf et al. (2021)]. Root exudates were collected into fresh trap solution for 4 h and secondary metabolites were extracted via liquid-phase extraction as described above. Samples were then analyzed for vanillin via HPLC-PDA at 280 nm (Accela HPLC/PDA, Thermo Scientific, Waltham, United States) using a Kinetex 2.6 μm XB-C18 100A reverse phase column (Phenomenex, Torrance, United States) with formate buffer (10 mM, pH 3.7) as polar and acetonitrile as nonpolar eluent (flow rate 0.5 ml·min^−1^), and a commercial standard (Aldrich Chem. Co., Inc., Milwaukee, United States) in the range from 0.25–2.0 mg·L^−1^. The applied eluent gradients are provided in the Supplementary Table S5 (Supplementary Material).

### 2.7 Statistics

Statistical analysis was performed, and plots were created with R version 3.5.3 (R Core Team, 2018) using the packages “lsmeans,” “multcompView” and “ggplot2.” “lsmeans” package was used to perform an ANOVA and “multcompView” to evaluate statistical significance of the assessed treatments combinations on vanillin exudation rates using Tukey-Tests. Package “ggplot2” was used to create the figures.

## 3 Results

### 3.1 Isolation and identification of secondary metabolites

**FIGURE 1:**
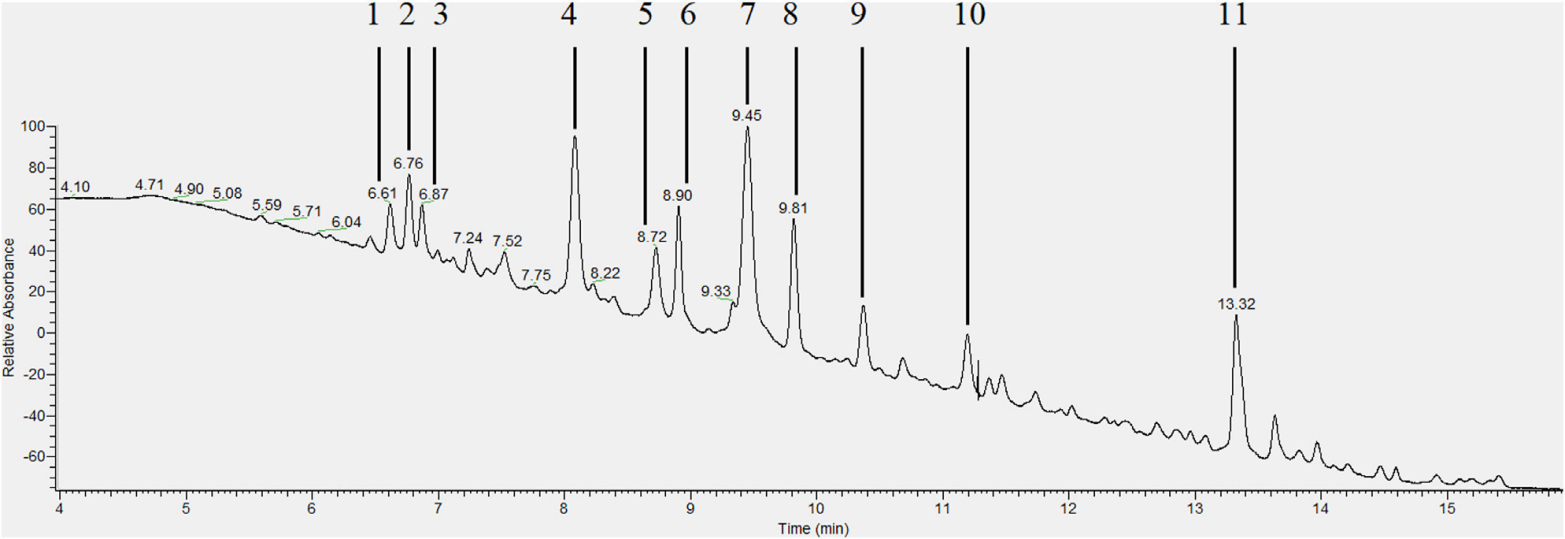
HPLC-PDA chromatogram (wavelength range 200–600 nm) of the extracted root exudates of *Brachiaria humidicola* with peaks corresponding to isolated fractions [adapted from Egenolf et al. (2020)].

**FIGURE 2:**
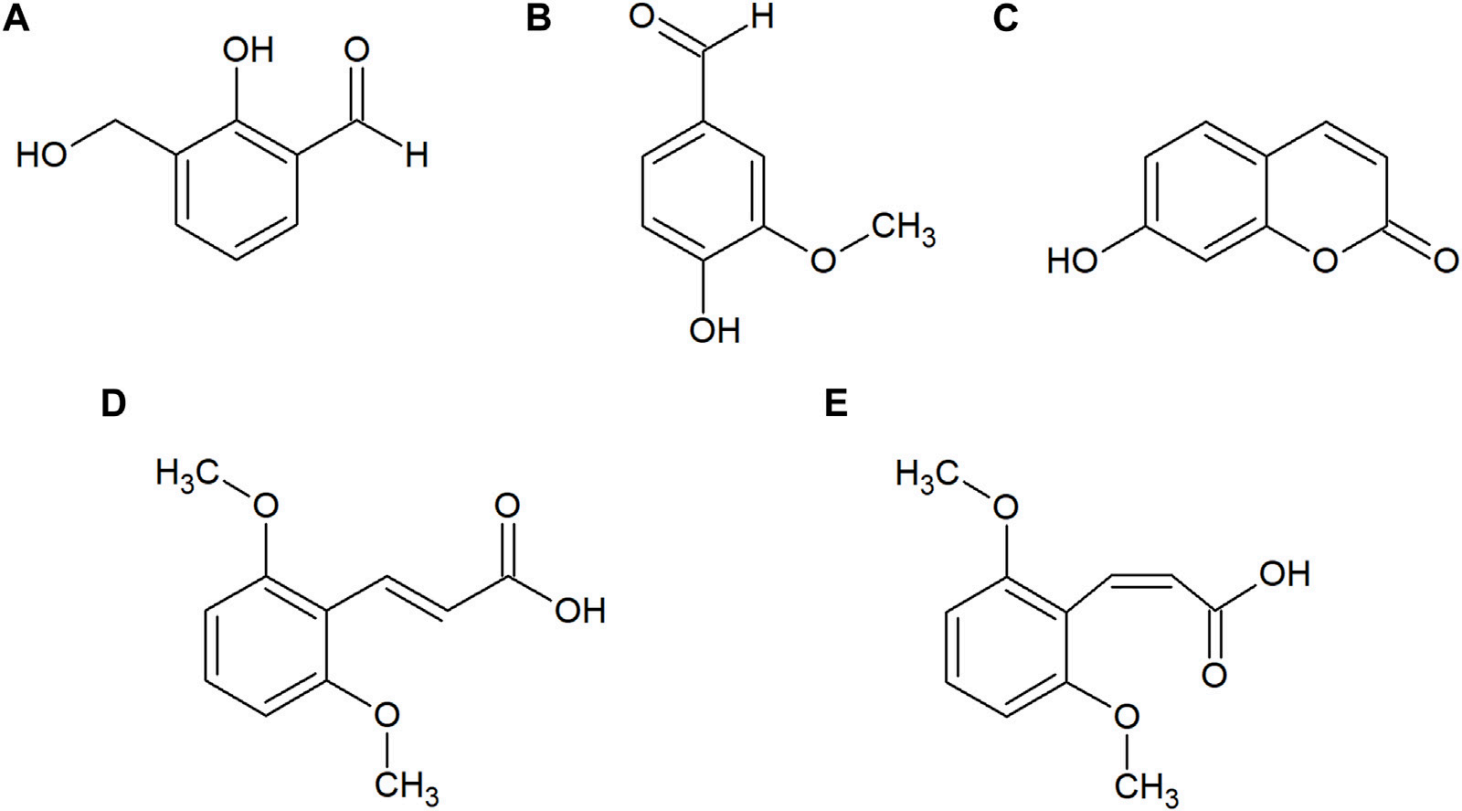
Chemical structures of 2-hydroxy-3-(hydroxymethyl)benzaldehyde **(A)**, vanillin **(B)**, umbelliferone **(C)**, *trans*-2,6-dimethoxycinnamic acid **(D)** and *cis*-2,6-dimethoxycinnamic acid **(E)** isolated from the root exudates of *B. humidicola*.

HPLC-PDA analysis of *B. humidicola* root exudates revealed eleven major peaks (Figure 1). At this stage, only few peaks showed distinct UV absorption spectra, indicating an overlay of several signals per peak. After fractionation of root exudates by semi-preparative HPLC and isolation of at least one compound per fraction, the major metabolites related to 7 out of 11 fractions were identified via HRMS and NMR spectroscopic techniques. Two fractions, namely, fractions 7 and 10, consisted of metabolites occurring in two different isomeric forms, totaling in 9 distinct secondary metabolites. The four cyclic diterpenes identified and classified as different brachialactone isomers and derivatives (fractions 4, 9 and 10) have been discussed earlier (Egenolf et al., 2020) and are not subject of this study. Remaining metabolites were classified as phenol and cinnamic acid derivatives. More precisely, the evaluation of HRMS, 1D and 2D NMR spectra identified fraction 1 (*m/z* 152) as 2-hydroxy-3-(hydroxymethyl)benzaldehyde (**a**), fraction 2 (*m/z* 152) as vanillin (**b**), fraction 3 (*m/z* 162) as umbelliferone (**c**) and fraction 7 (*m/z* 208) as a mixture of both *trans-* and *cis*-2,6-dimethoxycinnamic acid (**d** and **e**) (Figure 2). The HRMS recordings and the NMR-data confirming the chemical structures of these metabolites are provided in Supplementary Tables S6–S11 (Supplementary Material). The characterization of fractions 5, 6, 8 and 11 was not possible, due to impurity of the isolate, even after a second purification via semi-preparative HPLC.

### 3.2 Nitrification inhibitory potential of isolated fractions/metabolites

**FIGURE 3:**
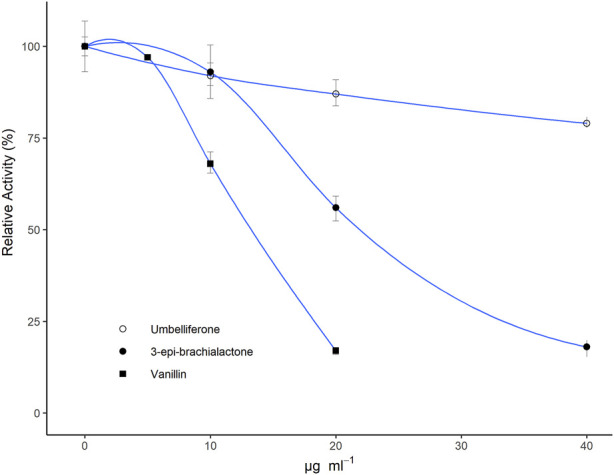
Relative metabolic activity (determined via bioluminescence measurements) of *Nitrosomonas europaea* IFO 14298 (ATCC 19178) pHLUX20 cultures in dependence of tested doses (10–40 μg·ml^−1^) of umbelliferone and vanillin in relation to previously assessed 3-*epi*-brachialactone (Egenolf et al., 2020). All metabolites were solubilized in DMSO and spiked to the assay medium. Results are displayed relative to a DMSO blank. Data represent the mean of three technical replications. Error bars represent standard errors (SE).

All isolated fractions or purified metabolites were assessed for their nitrification inhibiting activity in a *N. europaea* based liquid culture assay (Subbarao et al., 2006; Nuñez et al., 2018) at concentrations ranging from 0–40 μg·ml^−1^. Among all isolated fractions, vanillin (**b**, ED_50_ ∼ 12.5 μg·ml^−1^ ED_80_ ∼ 20 μg·ml^−1^) and the fractions 6 and 8 (ED_50_ ∼ 7.5–12.5 μg·ml^−1^ ED_80_ ∼ 15–25 μg·ml^−1^) showed the strongest inhibitory effects (Table 1). In contrast, only a slight inhibitory effect was detected for umbelliferone (**c**, ED_50_ ∼ 100 μg·ml^−1^), whereas 2-hydroxy-3-(hydroxymethyl)benzaldehyde (**a**) and both *trans*- and *cis*-2,6-dimethoxycinnamic acid (**d** and **e**) did not show any inhibitory, but a weak stimulatory effect on *N*. *europaea* (36%–46% at 20 μg·ml^−1^). The dose-response curves for vanillin (**b**), umbelliferone (**c**) and previously described 3-*epi*-brachialactone are displayed in Figure 3.

**TABLE 1:** Composition of *Brachiaria humidicola* root exudates and nitrification inhibitory activity of each fraction/metabolite.

Fraction	Retention time	Molecular mass	Metabolite	Yield	Exudation rate	Effect on the activity of *N. europaea* at 20 μg·ml^−1^	Effective dose	
							ED_50_	ED_80_
	min	g/mol		µg	µg·h^−1^·g^−1^ root DM	% (S.D.)	µg·ml^−1^	
1	6.61	152	2-Hydroxy-3-(hydroxymethyl) benzaldehyde	410		+46 (3)		
2	6.78	152	**Vanillin**	100	1–2	−83 (1)	**12.5**	**20**
3	6.87	162	Umbelliferone	240	3–6	−13 (6)	100	-
4	8.08	350	16-Hydroxy-3-*epi*-brachialactone	110		+41 (9)		
5	8.72	243 ?	n.d.	-		n.d.		
6	8.90	Several compounds	n.d.	400		−83 (3)	7.5	15
7	9.45	208	*trans*-2,6-Dimethoxy cinnamic acid	300		+36 (15)		
		208	*cis*-2,6-Dimethoxy cinnamic acid					
8	9.81	Several compounds	n.d.	200		−76 (2)	12.5	25
9	10.37	332	3,18-Epoxy-9-hydroxy-4,7-*seco*-brachialactone	170		−19 (2)	40	
10	11.20	334	Brachialactone	180		+17 (9)		
		334	**3-*epi*-Brachialactone**	160	1–4	−44 (6)	**20**	**40**
11	13.32	278 ?	n.d.	-		n.d.		

Yield refers to the amount of purified metabolite isolated from approximately 100 L of nutrient solution (400 plants). Exudation rates were quantified in a hydroponic system, nitrification inhibiting (NI) activity through a bioassay using pure cultures of *Nitrosomonas europaea* IFO 14298 (ATCC 19178) pHLUX20. Bold values are the main NI detected for B.h.

### 3.3 Vanillin exudation in dependence of rhizosphere pH and nutritional N-form

**FIGURE 4:**
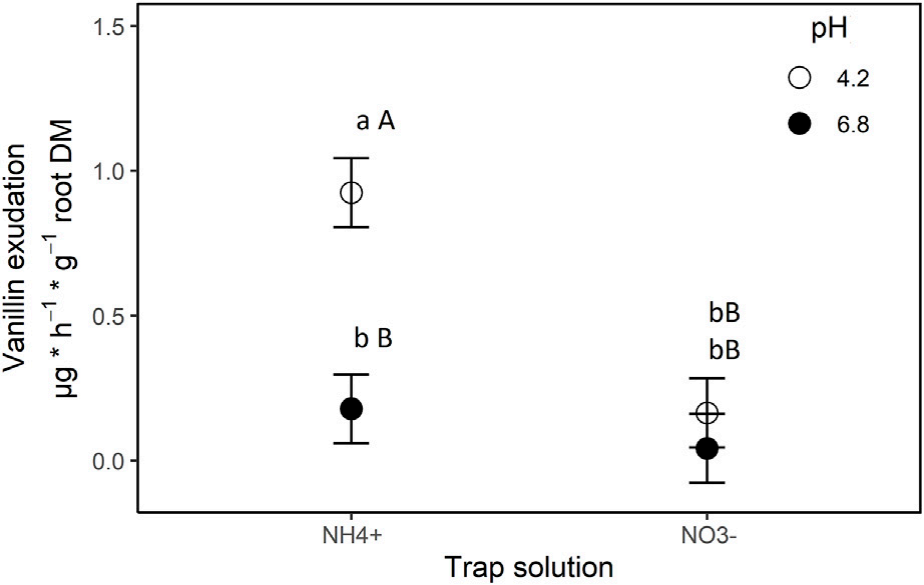
Exudation of vanillin by roots of *B. humidicola* into trap solutions differing in nitrogen source (*x*-axis) and pH (legend). Trap solutions were complete nutrient solutions with nitrogen being offered as ammonium (NH_4_^+^ treatment) or nitrate (NO_3_^−^ treatment). Results represent least square means of four biological replications, error bars indicate standard error (SE) of the mean. Different uppercase letters indicates statistical difference for least-square means (*α* = 0.05) between N-treatments, lowercase letters indicate statistical differences between pH-treatments (*α* = 0.05).

Both experimental factors had a significant effect on exudation rates of vanillin (N form: *p* = 0.0001; pH: *p* = 0.0336; N form*pH: *p* = 0.043). Especially the combination of NH_4_^+^ nutrition and low pH (4.2) prompted vanillin exudation (Figure 4), a finding in accordance with previous reports on NI exudation patterns in general (Subbarao et al., 2007b; Subbarao et al., 2009) and on 3-*epi*-brachialactone in specific (Egenolf et al., 2021).

## 4 Discussion

In this study, root exudates of *B. humidicola* were screened for novel nitrification inhibiting secondary metabolites. Besides the previously described NI brachialactone (Subbarao et al., 2009) and 3-*epi*-brachialactone (Egenolf et al., 2020), three additional fractions, namely, fraction 2 (vanillin), 6 and 8, possessed strong and substantially higher nitrification inhibiting activity (ED_80_ = 15–25 μg·ml^−1^) than both brachialactone isomers (Egenolf et al., 2020). With this, our hypothesis that the nitrification inhibiting activity of root exudates of *B. humidicola* is not only founded in the presence of brachialactones, but is also facilitated by additional NI (i.e., vanillin), could be verified.

Vanillin is the main flavoring agent of natural vanilla. In *Vanilla* spp., it occurs as vanillin-*β*-D-glucoside, with the aromatic aglycon vanillin accumulating to concentrations of 2%–2.5% after curing of pods. This phenolic aldehyde has also been described for a variety of crops, e.g., coffee, strawberries, tobacco, and grapes (Demian, 1993). The biosynthetic pathway of vanillin is linked to biosynthesis of lignin, one of the key structural polymers in plants. More precisely, two alternative biosynthetic pathways have been evidenced (Walton et al., 2003). The first pathway was proposed by Zenk (1965), suggesting β-oxidation of feruloyl-CoA to vanilloyl-CoA (analogous to fatty acid β-oxidation) and subsequent reduction to vanillin. The second pathway was proposed by Kanisawa et al. (1994), suggesting that vanillin-*β*-D-glucoside may arise directly from *p*-coumaric acid via *p*-hydroxybenzaldehyde (Podstolski et al., 2002; Walton et al., 2003). The link to lignin metabolism is obvious, as the common precursor to both vanillin biosynthetic pathways—*p*-coumaric acid—constitutes the central intermediate in the biosynthesis of the three monolignols *p*-coumaryl alcohol (principal monolignol in grasses), coniferyl alcohol (principal monolignol in gymnosperms) and sinapyl alcohol (Boerjan et al., 2003).

The biosynthetic pathway of vanillin via *p*-coumaric and/or ferulic acid deserves attention, as all three metabolites have been reported to possess an allelopathic potential (Singh et al., 2001; Reigosa and Malvido-Pazos, 2007), including herbicidal effects (Chuah et al., 2013). In leaf and root tissues of *B. humidicola*, various allelopathic metabolites have been identified. These comprise brachialactol, different flavones (especially quercetin glycosides) and saponins, but especially different phenolic acids, i.e., *p*-coumaric acid, *p*-hydroxy-benzoic acid and vanillic acid (Supplementary Table S12; Supplementary Material). These allelopathica have been proposed to be responsible for the suppression of companion plants (e.g., grass-legume mixtures) and responsible for the dominance of *B. humidicola* in many ecosystems (Souza Filho et al., 2005; Oliveira et al., 2017; Feitoza et al., 2018; Feitoza et al., 2020). However, with regard to nitrification inhibition, none of the discussed phenolic acids (*p*-coumaric acid, ferulic acid, *p*-hydroxy-benzoic acid, vanillic acid) showed inhibitory activity against *N. europaea*, which was tested *in vitro* with concentrations up to 100 mg·L^−1^ (data not shown). These results are in line with *in vitro* and soil incubation studies on the nitrification inhibitory potential of *p*-coumaric acid and ferulic acid by McCarty et al. (1991) and Wu et al. (1999), contradicting initial findings by Rice and Pancholy (1974). In contrast, different methyl-derivatives of these phenolic acids, namely, methyl coumarate and methyl ferulate (isolated from *B. humidicola* roots) and methyl 3-(4-hydroxyphenyl) propionate (a root exudate of *Sorghum bicolor*) have been verified as biological NI (Gopalakrishnan et al., 2007; Zakir et al., 2008). Considering that this pattern resembles the case of vanillic acid and vanillin (only the latter revealed a nitrification inhibiting activity, ED_80_ ∼ 20 μg·ml^−1^), it could be deduced that the biological activity of these phenolics is determined by the carboxylic acid/aldehyde functional group.

At present, it remains impossible to estimate the individual contribution of each of the described metabolites to the overall allelopathic and nitrification inhibiting activity identified for *B. humidicola*. This is mainly related to the lack of data on internal concentrations and release rates through root turnover or active exudation *in situ*, as well as an insufficient understanding of their persistence and especially activity in soils. In this regard, data on root tissue concentrations have been provided for 3-*epi*-brachialactone [2–8 μg·g^−1^ root DM (Egenolf et al., 2021)], but not for vanillin (or possible vanillin glycosides), although the presence of all precursors strongly suggests internal vanillin pools (Oliveira et al., 2017). With regard to NI release, both 3-*epi*-brachialactone and vanillin exudation rates have been quantified in the same range of 1–4 μg·h^−1^·g^−1^ root DM (hydroponic studies) and to depend on external pH and cation feeding, suggesting active release via secondary transporters [see Figure 4 of this article and Egenolf et al. (2021)]. Whether the encountered exudation rates are sufficient to induce an accumulation of bioactive NI in soils, remains however to be addressed in subsequent studies, emphasizing that *in situ* exudation rates often lie several magnitudes higher than those observed in artificial systems (G. Neumann, personal communication). Furthermore, the allelopathic control of soil nitrification through the described putative NI, still has to be proven *in situ*, one possible approach constituting the simultaneous assessment and subsequent correlation of soil nitrification rates with the rhizosphere concentrations of the respective compounds.

In relation to the verification of the encountered effects within soils and considering the exclusion of any detrimental impact on the soil microbiome, the discussed phenolics might represent potential candidates responsible for BNI activity of *B. humidicola*. Especially the hypothesized metabolic pathways (1) phenylalanine → *p*-coumaric acid (→ methyl coumarate) → 4-hydroxybenzaldehyde → 3,4-dihydroxybenzaldehyde → vanillin as well as (2) phenylalanine → caffeic acid → ferulic acid (→ methyl ferulate) → vanilloylCoA → vanillin deserve further attention with respect to BNI breeding, including *B. humidicola* and other crops (Walton et al., 2003).

When breeding is concerned, potential co-benefits and trade-offs of the discussed phenolics with general allelopathic/antibiotic potentials must be considered. These comprise 1) potential non-target effects on the soil microbiome beyond ammonia oxidizers (e.g., poor N mineralization in extensive pasture systems), 2) allelopathic suppression of companion legumes within the pasture system, 3) feed quality in general (palatability), 4) harmful effects on animal health as some phenolic provoke secondary photosensitization of ruminants (Oliveira et al., 2017), and 5) effects on ruminal methane emissions reported for tannin-rich feeds (Verma et al., 2021).

## Data Availability

The original contributions presented in the study are included in the article/Supplementary Material, further inquiries can be directed to the corresponding author.
