# Supplementary material for: Root exudate fingerprint of Brachiaria humidicola reveals vanillin as a novel and effective nitrification inhibitor
Source: Front Mol Biosci. 2023 Dec 5;10:1192043. doi: 10.3389/fmolb.2023.1192043 (PMC10728723; doi:10.3389/fmolb.2023.1192043)
Supplement: Supplementary file 1 [file Table1.docx]

***Supplementary Material***

**Root exudate fingerprint of *Brachiaria humidicola* reveals vanillin as a novel and effective nitrification inhibitor**

Konrad Egenolf^1,2^, Jochen Schöne^3^, Jürgen Conrad^4^, Christina Braunberger^4^, Uwe Beifuß^4^, Jacobo Arango^2^, Frank Rasche^1*†^

^1^Institute of Agricultural Sciences in the Tropics (Hans-Ruthenberg-Institute), University of Hohenheim, 70593 Stuttgart, Germany

^2^Tropical Forages Program, The Alliance of Bioversity International and the International Center for Tropical Agriculture (CIAT), Cali, Colombia

^3^Institute of Phytomedicine, University of Hohenheim, 70593 Stuttgart, Germany

^4^Institute of Chemistry, University of Hohenheim, 70593 Stuttgart, Germany

^†^Present address: International Institute of Tropical Agriculture (IITA), PO Box 30772-00100, Nairobi, Kenya

***Correspondence:**

Prof. Dr. Frank Rasche

Email: [frank.rasche@uni-hohenheim.de](mailto:frank.rasche@uni-hohenheim.de)

**Table S1.** Gradient HPLC-PDA. Column: Phenomenex Kinetex 2.6µ XB-C18 100A, flow rate: 0.5 ml min^-1^.

| Time (min) | % nonpolar eluent  (acetonitrile) | % polar eluent  (10mM formate buffer) |
| --- | --- | --- |
| 0.00 | 15 | 85 |
| 1.00 | 15 | 85 |
| 11.00 | 100 | 0 |
| 13.00 | 100 | 0 |
| 13.10 | 15 | 85 |
| 16.00 | 15 | 85 |

**Table S2.** Gradient 1. semi-preparative HPLC separation. Column: *Macherey-Nagel EC 250/10 Nucleodur PolarTec 5µm*, flow rate: 5.0 ml min^-1^.

| Time (min) | % nonpolar eluent  (acetonitrile) | % polar eluent  (0.01% TFA) |
| --- | --- | --- |
| 0.00 | 15 | 85 |
| 2.00 | 15 | 85 |
| 56.00 | 55 | 45 |
| 92.00 | 100 | 0 |
| 98.00 | 100 | 0 |
| 100.00 | 15 | 85 |

**Table S3.** Gradient 2. semi-preparative HPLC separation. Column: *Waters xSelect HSS Prep T3 5µm 10x150mm*, flow rate 5.0 ml min^-1^.

| Time (min) | % nonpolar eluent (100% acetonitrile) | % polar eluent  (0.01% TFA) |
| --- | --- | --- |
| 0.00 | 40 | 60 |
| 3.00 | 40 | 60 |
| 21.00 | 45 | 55 |
| 24.00 | 100 | 0 |
| 28.00 | 100 | 0 |
| 30.00 | 40 | 60 |

**Table S4.** Gradient UPLC-HRMS. Column: *Waters AQUITY UPLC HSS 1.8µm 2.1x150mm*, 40°C, flow rate 0.35 ml min^-1^.

| Time (min) | % nonpolar eluent (acetonitrile, 0.2% formic acid) | % polar eluent  (0.01% Formic Acid) |
| --- | --- | --- |
| 0.00 | 20 | 80 |
| 5.00 | 25 | 75 |
| 20.00 | 60 | 40 |
| 30.00 | 95 | 5 |
| 35.00 | 95 | 5 |
| 36.00 | 20 | 80 |
| 39.00 | 20 | 80 |

**Table S5.** Gradient analytical HPLC-PDA for vanillin quantification. Column: Phenomenex Kinetex 2.6μ XB-C18 100A, flow rate: 0.5 ml min^-1^.

| Time  (min) | % nonpolar eluent  (acetonitrile) | % polar gradient  (10mM formate buffer) |
| --- | --- | --- |
| 0.00 | 10 | 90 |
| 1.00 | 10 | 90 |
| 15.00 | 90 | 10 |
| 17.00 | 90 | 10 |
| 17.10 | 10 | 90 |
| 20.00 | 10 | 90 |

**Table S6.** HRMS recordings of selected fractions of *Brachiaria humidicola* root exudates.

| Fraction | Retention time | Scan | Molecular mass [M+H]+ | Theoretical mass [M+H]+ | Delta | Composition | Metabolite |
| --- | --- | --- | --- | --- | --- | --- | --- |
|  | min |  | *m/z* | *m/z* | mmu |  |  |
| **1** | 6.61 | - | 151.03916 | 151.04007 | -0.91 | C_8_H_7_O_3_ | 2-Hydroxy-3-(hydroxymethyl) benzaldehyde |
| **2** | 6.78 | - | 151.03911 | 151.04007 | -0.96 | C_8_H_7_O_3_ | Vanillin |
|  |  | + | 153.05483 | 153.05462 | 0.21 | C_8_H_9_O_3_ |  |
| **3** | 6.87 | - | 161.02355 | 161.02442 | -0.87 | C_9_H_5_O_3_ | Umbelliferone |
|  |  | + | 163.03913 | 163.03897 | 0.16 | C_9_H_7_O_3_ |  |
| **4** | 8.08 | + | 351.21689 | 351.21660 | 0.29 | C_20_H_31_O_5_ | 16-Hydroxy-3-*epi*-brachialactone |
| **5** | 8.72 | n.d. | | | | | |
| **6** | 8.90 | n.d. | | | | | |
| **7** | 9.45 | + | 209.08093 | 209.08084 | 0.09 | C_11_H_13_O_4_ | *trans/*-2,6-Dimethoxy cinnamic acid |
|  |  | + | 209.08093 | 209.08084 | 0.09 | C_11_H_13_O_4_ | *cis*-2,6-Dimethoxy cinnamic acid |
| **8** | 9.81 | n.d. | | | | | |
| **9** | 10.37 | + | 333.20612 | 333.20604 | 0.08 | C_20_H_29_O_4_ | 3,18-Epoxy-9-hydroxy-4,7-*seco*-brachialactone |
| **10** | 11.20 | + | 335.22171 | 335.22169 | 0.02 | C_20_H_31_O_4_ | Brachialactone |
|  |  | + | 335.22171 | 335.22169 | 0.02 | C_20_H_31_O_4_ | 3‑*epi*-Brachialactone |
| **11** | 13.32 | n.d. | | | | | |

**Table S7**. NMR-spectral analysis of 2-hydroxy-3-(hydroxymethyl)benzaldehyde (**1**) in MeOH-*d_4_*.

| Atom | δH (ppm), multiplicity, *J* (Hz) | δC (ppm) | HMBC |  |
| --- | --- | --- | --- | --- |
| 1 | - | 121.5 |  | 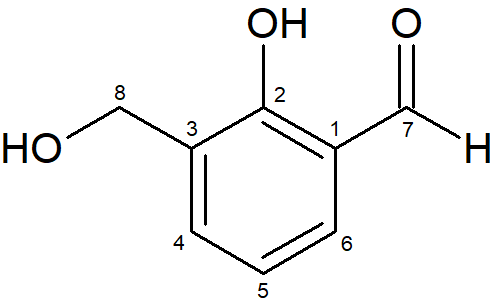 |
| 2 | - | 159.6 |  |  |
| 3 | - | 130.8 |  |  |
| 4 | 7.73 ddt (7.5, 1.67, 0.9) | 136.1 | C2, C8, C6 |  |
| 5 | 7.11 dd (7.6, 7.6) | 120.4 | C1, C3 |  |
| 6 | 7.66 dd (1.6, 7.6) | 133.7 | C2, C7 |  |
| 7 | 9.99 s | 198.5 | C1, C2 |  |
| 8 | 4.73 s | 58.7 | C2, C3, C4 |  |

**Table S8**. NMR-spectral analysis of vanillin (**2**) in MeOH-*d_4_*.

| Atom | δH (ppm), multiplicity, *J* (Hz) | δC (ppm) | HMBC |  |
| --- | --- | --- | --- | --- |
| 1 | - | 130.3 |  | 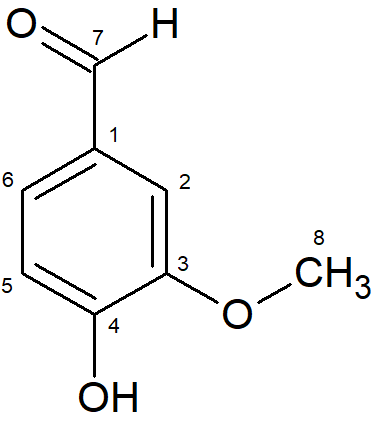 |
| 2 | 7.49 d (1.75) | 111.6 | C4, C6 |  |
| 3 | - | 149.3 |  |  |
| 4 | - | 154.3 |  |  |
| 5 | 6.98 d (8.12) | 116.2 | C1, C3 |  |
| 6 | 7.47 dd (7.7, 1.87) | 127.9 | C4 |  |
| 7 | 9.79 | - | C1 |  |
| 8 | 3.96 s | 56.2 | C3 |  |

**Table S9**. NMR-spectral analysis of umbelliferone (**3**) in MeOH-*d_4_*.

| Atom | δH (ppm), multiplicity, *J* (Hz) | δC (ppm) | HMBC |  |
| --- | --- | --- | --- | --- |
| 1 | - | - |  | 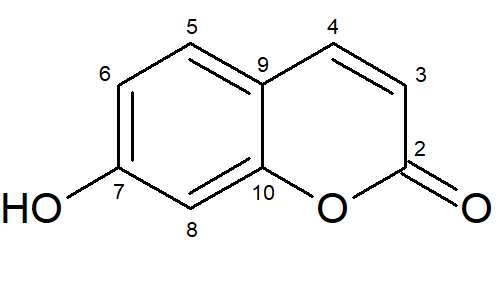 |
| 2 | - | 163.7 |  |  |
| 3 | 6.22 d (9.5) | 111.9 | C2, C9 |  |
| 4 | 7.89 d (9.5) | 145.9 | C2, C5, C10 |  |
| 5 | 7.49 d (8.6) | 130.6 | C4, C7, C10 |  |
| 6 | 6.83 dd (2.2, 8.6) | 114.2 | C8 |  |
| 7 | - | 163.2 |  |  |
| 8 | 6.75 d (2.2) | 103.3 | C9 |  |
| 9 | - | 113.0 |  |  |
| 10 | - | 157.0 |  |  |

Coupling constants were directly taken form the NMR spectrum and were not averaged.

Abbreviations: s = singlet, d = doublet, dd = doublet of doublets, ddd = doublet of doublet of doublets, t = triplet, dt = doublet of triplets, q = quartet, m = multiplet, br = broad, ax = axial, eq = equatorial, ov = overlapped with other signal, n.o. = not observable.

**Table S10**. NMR-spectral analysis of *trans*-2,6-dimethoxycinnamic acid (**4**) in MeOH-*d_4_*.

| Atom | δH (ppm), multiplicity, *J* (Hz) | δC (ppm) | HMBC |  |
| --- | --- | --- | --- | --- |
| 1 | - | 112.8 |  | 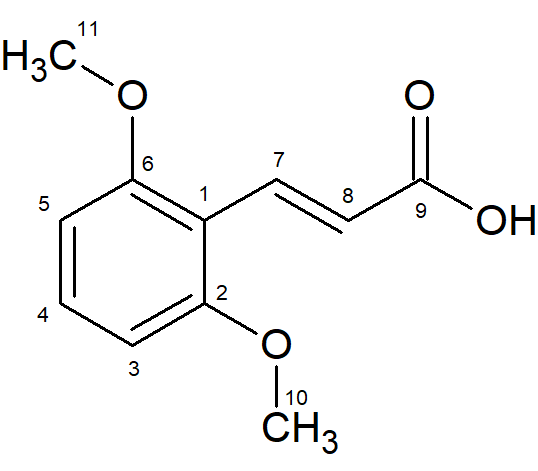 |
| 2 | - | 161.2 |  |  |
| 3 | 6.72 d (8.4) | 104.8 | C5 |  |
| 4 | 7.36 t (8.4) | 132.9 | C2 |  |
| 5 | - | - |  |  |
| 6 | - | - |  |  |
| 7 | 8.15 d (16.5) | 137.4 | C2, C8, C9 |  |
| 8 | 6.85 brd (16.5) | 120.9 |  |  |
| 9 |  | 171.9 |  |  |
| 10 | 3.94 s | 56.1 | C2 |  |
| 11 |  | - |  |  |

**Table S11**. NMR-spectral analysis of *cis*-2,6-dimethoxycinnamic acid (**4**) in MeOH-*d_4_*.

| Atom | δH (ppm), multiplicity, *J* (Hz) | δC (ppm) | HMBC |  |
| --- | --- | --- | --- | --- |
| 1 | - | 114.8 |  | 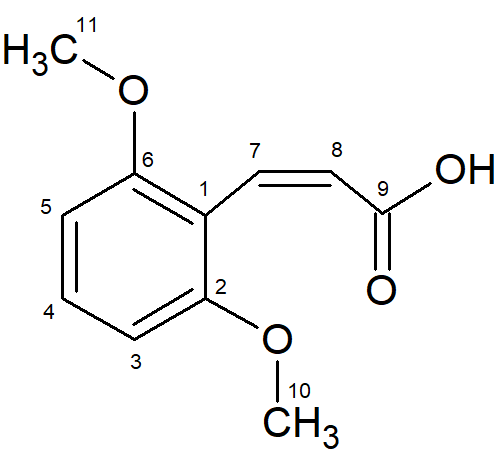 |
| 2 | - | 158.8 |  |  |
| 3 | 6.64 d (8.4) | 104.6 |  |  |
| 4 | 7.27 t (8.4) | 130.8 | C2 |  |
| 5 | - | - | C1, C3 |  |
| 6 | - | - |  |  |
| 7 | 6.84 d (12.1) | 134.2 | C2 |  |
| 8 | 6.02 brd (12.1) | 123.3 |  |  |
| 9 |  | - |  |  |
| 10 | 3.82 s | 55.7 | C2 |  |
| 11 |  | - |  |  |

Coupling constants were directly taken form the NMR spectrum and were not averaged.

Abbreviations: s = singlet, d = doublet, dd = doublet of doublets, ddd = doublet of doublet of doublets, t = triplet, dt = double of triplets, q = quartet, m = multiplet, br = broad, ax = axial, eq = equatorial, ov = overlapped with other signal, n.o. = not observable.

**Table S12:** Putative allelopathic compounds described for *Brachiaria humidicola*

| Compound Class | Compound | Origin | Approximated tissue concentration /  Exudation rates | Source |
| --- | --- | --- | --- | --- |
| Phenolics | *p*-Hydroxybenzoic acid | Leaf tissue | ~ 20 µg g^-1^ | (Oliveira et al., 2017) |
|  | 2-Hydroxy-3-(hydroxymethyl) benzaldehyde | Root exudates |  |  |
|  | Vanillic acid | Leaf tissue | ~ 20 µg g^-1^ | (Oliveira et al., 2017) |
|  | Vanillin | Root exudates |  |  |
|  | *trans/cis*-2,6-Dimethoxycinnamic acid | Root exudates |  |  |
|  | *p*-Coumaric acid | Leaf tissue | 3.5 -20 µg g^-1^ | (Souza Filho et al., 2005; Oliveira et al., 2017) |
|  | Methyl coumarate | Root tissue |  | (Gopalakrishnan et al., 2007) |
|  | Methyl ferulate | Root tissue |  | (Gopalakrishnan et al., 2007) |
|  | Umbelliferone | Root exudates |  |  |
| Flavonols | Isorhamnetin-3-*O*-*β*-D-glucopyranoside, methyl-quercetin-3-*O-β*-D-glucuronate, quercetin-3-*O-α*-L-rhamnopyranoside, tricin, kaempferitrin | Leaf tissue |  | (Oliveira et al., 2017) |
| Saponins | Dioscin, Penogenin-3-*O*-*α*-L-rhamnopyranosyl-  (1-4)-[*α*-l-rhamnopyranosyl-(1-2)]*-β*-D-glucopyranosyl, catechin-7-*O-β*-D-glucopyranoside, Floribundasaponin B, Humidicine A-D | Root tissue |  | (Oliveira et al., 2017; Feitoza et al., 2020) |
| Terpenoids | Brachialactol, 16-hydroxy-3-*epi*-brachialactone, 3-*epi*-brachialactone | Root tissue | ~ 4 µg g^-1^ (brachialactol)  ~ 2-8 µg g^-1^ (3-*epi*-brachialactone) | (Egenolf et al., 2020; Feitoza et al., 2020) |
|  | Brachialactone, 3-*epi*-brachialactone, 3,18-epoxy-9-hydroxy-4,7-*seco*-brachialactone | Root exudates | 0.4 - 4.0 µg h^-1^ g ^-1^ (Brachialactone & 3-*epi*-brachialactone) | (Subbarao et al., 2009; Egenolf et al., 2021) |
